# Lower Levels of ABO Anti-A and Anti-B of IgM, IgG and IgA Isotypes in the Serum but Not the Saliva of COVID-19 Convalescents

**DOI:** 10.3390/jcm11154513

**Published:** 2022-08-02

**Authors:** Eva M. Matzhold, Günther F. Körmöczi, Chiara Banfi, Marlies Schönbacher, Camilla Drexler-Helmberg, Ivo Steinmetz, Andrea Berghold, Peter Schlenke, Gabriel E. Wagner, Anja Stoisser, Barbara Kleinhappl, Wolfgang R. Mayr, Thomas Wagner

**Affiliations:** 1 Department of Blood Group Serology and Transfusion Medicine, Medical University of Graz, 8036 Graz, Austria; camilla.drexler@uniklinikum.kages.at (C.D.-H.); peter.schlenke@medunigraz.at (P.S.); anja.stoisser@medunigraz.at (A.S.); thomas.wagner@medunigraz.at (T.W.); 2 Department of Blood Group Serology and Transfusion Medicine, Medical University of Vienna, 1090 Vienna, Austria; guenther.koermoeczi@meduniwien.ac.at (G.F.K.); marlies.schoenbacher@meduniwien.ac.at (M.S.); wolfgang.mayr@meduniwien.ac.at (W.R.M.); 3 Institute for Medical Informatics, Statistics and Documentation, Medical University of Graz, 8036 Graz, Austria; chiara.banfi@medunigraz.at (C.B.); andrea.berghold@medunigraz.at (A.B.); 4 Diagnostic & Research Institute of Hygiene, Microbiology and Environmental Medicine, Diagnostic and Research Center for Molecular Biomedicine, Medical University of Graz, 8010 Graz, Austria; ivo.steinmetz@medunigraz.at (I.S.); gabriel.wagner-lichtenegger@medunigraz.at (G.E.W.); barbara.kleinhappl@medunigraz.at (B.K.)

**Keywords:** SARS-CoV-2, COVID-19, ABO blood group, ABO antibodies, immune system, immunoglobulin A, saliva

## Abstract

Individuals with ABO type O, naturally possessing anti-A and anti-B antibodies in their serum, are underrepresented among patients infected with SARS-CoV-2 compared with healthy controls. The ABO antibodies might play a role in the viral transmission. Therefore, we aimed to quantify anti-A/anti-B, including their subclasses IgM, IgG and IgA, in the serum and saliva of Caucasians (*n* = 187) after mild COVID-19 to compare them with individuals who had never been infected with SARS-CoV-2. Two samples were collected within two months after the diagnosis (median days: 44) and two months later. ABO antibodies were determined by flow cytometry. Additionally, total IgA in saliva and antibodies specific to SARS-CoV-2 were tested by ELISA. COVID-19 convalescents had significantly lower levels of anti-A/anti-B IgM, IgG and IgA in their serum than control subjects (*p* < 0.001). Interestingly, no significant differences were observed in saliva. ABO antibody levels remained stable over the period considered. No relation of ABO to the level of SARS-CoV-2-specific antibodies was observed. Total IgA was lower in convalescents than in controls (*p* = 0.038). Whereas ABO antibodies in the saliva may not contribute to the pathogenesis of COVID-19, individual pre-existing high serum concentrations of anti-A/anti-B may have a protective effect against SARS-CoV-2 infection.

## 1. Introduction

In patients with coronavirus disease 2019 (COVID-19), a lower proportion of individuals with ABO blood type O [1,2,3], accompanied by a higher proportion of type AB [4,5,6], was reported when compared with healthy controls. Whereas individuals with blood type AB do not possess ABO antibodies in their serum, blood type O is associated with both anti-A and anti-B antibodies. Since no interactions of the ABO and secretor types were observed in our own preceding study [6], we hypothesized that the variable susceptibility to infection with severe acute respiratory syndrome coronavirus 2 (SARS-CoV-2) might be related to interference caused by circulating ABO antibodies (also called ABO isoagglutinins). In this regard, the inhibitory effects of anti-A on infectivity of SARS-CoV-1 were described in vitro [7]. Whether they play a key role in the underlying pathophysiology of ABO blood groups and COVID-19 was discussed [8].

Recent investigations demonstrated the adhesion of the SARS-CoV-2 receptor binding domain to the A antigen on a solid-phase glycan microarray [9]. Further, the adhesion glycoprotein was described as expressing ABO antigens when produced during viral replication in individuals with a non-O blood type [10]. It was predicted that SARS-CoV-2 binds to host cells via the formation of hybrid antigenic structures [11]. Composed of serologically A-like *N*-acetylgalactosamine glycan structures and the viral serine molecule, these might function as a host–pathogen molecular bridge. This does not challenge the established function of angiotensin-converting enzyme 2 (ACE2) receptor’s protein-based activity as previously described [12,13] but likely points to a mechanism by which ABO antibodies might interfere with the virus.

The naturally occurring anti-A and anti-B antibodies in the serum consist mainly of immunoglobulin (Ig) M and, to a lesser extent, IgG [14]. Divergent results regarding ABO antibody levels among different populations and ethnicities have been observed [15,16,17,18]. Large variations in anti-A and anti-B titres in the serum between individuals of the same blood type are well known; however, antibody levels remain stable over time [19,20]. Each immunoglobulin of the IgM, IgG and IgA isotypes has a unique profile of effector functions capable of mediating host defence against invading pathogens [21]. Immunoglobulin class A has an important role in mediating adaptive and humoral immune responses to mucosal surfaces, including the respiratory epithelium [22,23]. Since SARS-CoV-2 primarily affects the mucous membranes of the respiratory tract [24,25], we examined a potential relationship between the total IgA secreted in saliva (total IgA^sal^) and anti-A-/anti-B-specific IgA. We therefore analysed both parameters in parallel in saliva specimens.

A possible association between SARS-CoV-2-specific antibodies and ABO blood types was previously investigated. It was found that patients with blood types O and B had lower SARS-CoV-2 antibody titres than patients with blood types A and AB [26,27]. A dataset of the ABO blood type distribution in COVID-19 patients published by Zhao et al. [28] was re-analysed by Gerard et al. from the perspective of ABO antibodies instead of ABO blood group antigens [29]. Their results suggested that subjects with anti-A in their serum (blood types B and O) are significantly less represented in the COVID-19 group than those lacking anti-A antibodies (types A and AB). However, the ABO antibodies were predicted based on the ABO antigen status and not directly measured in the blood of the study participants. Recently, Deleers et al. reported significantly lower mean agglutination scores attributable to lower IgM anti-A and IgM anti-B titres in the serum of COVID-19 patients than in controls [10]. They also provided evidence that A and B antigens are expressed in lung epithelial cells, where infectious viral particles are likely to be produced.

In light of these observations, we hypothesized that natural anti-A and/or anti-B might play a protective role against SARS-CoV-2 infection when transmitted between individuals with different ABO blood types [30,31]. Assuming that ABO antibody activity in saliva reflects its activity in the respiratory mucosa, differences in ABO antibody concentrations in saliva were also expected. Therefore, in this prospective study, we aimed to investigate ABO antibody levels, including IgM, IgG and IgA isotypes, in the serum and saliva of non-hospitalised patients who recovered from mild COVID-19 and to compare them with those of individuals who had never been infected with the virus. We observed consistently lower serum concentrations of anti-A/anti-B in COVID-19 convalescents than in controls, possibly indicating a protective role of ABO antibodies in infection with SARS-CoV-2.

## 2. Materials and Methods

### 2.1. Convalescent COVID-19 Study Participants

Caucasian individuals (*n* = 192) who previously tested positive for SARS-CoV-2 RNA and who were identified as “recovered from COVID-19” after exemption by the authorities were included [32]. The participants were recruited between June 2020 and February 2021. Clinical symptoms of COVID-19 and demographic characteristics were recorded via an electronic questionnaire.

Blood and saliva collected at the baseline visit (V) after the remission of SARS-CoV-2 infection (V1: ≤84 days post-diagnosis) and 2 months later (V2) were investigated. Saliva samples were available at V1 for *n* = 157 (82%). Therefore, the amount of available data for serum is generally higher than that for saliva. The sample size is reported for each comparison in the respective tables mentioned in the results. The antibody response to SARS-CoV-2 was assessed with the pan-Ig Anti-SARS-CoV-2 S assay (Roche Diagnostics, Rotkreuz, Switzerland) as previously described [32]. Individuals who had been hospitalized with COVID-19 or who were pregnant were excluded from the study.

### 2.2. Control Groups

Control serum samples were preserved serum samples (−70 °C) from regular whole-blood donors (serum controls, *n* = 195). In accordance with the time interval between the sampling of the COVID-19 convalescents at V1 and V2, two samples from the same donor (D) drawn at two different time points (D1 and D2) were analysed. The serum controls were collected during blood donations in 2017 and were matched for ABO type, age and sex with the convalescent COVID-19 samples.

Saliva samples from those who provided the abovementioned control serum samples were not available. Therefore, we obtained a second group of controls (saliva donors; *n* = 164) for the saliva studies, recruited at blood donation drives between May and September 2021. As with the sampling of COVID-19 convalescents at V1 and V2, samples were collected and analysed at a second time point (D2), two months after the initial saliva donation (D1). Individuals who had previously tested positive for SARS-CoV-2 or had been vaccinated against SARS-CoV-2 were excluded. This was confirmed by testing for the presence of SARS-CoV-2 antibodies in their blood using LIAISON SARS-CoV-2 S1/S2 IgG (DiaSorin S.p.A. 13040 Saluggia, Italy; REF 311450). Results were considered positive at a value of 15 AU/mL or more.

### 2.3. Preparation of Saliva Samples

Between 1 and 4 mL of saliva was collected into a saliva collection aid (Salimetrics, LLC, Carlsbad, CA 92008, USA) or into a 50 mL Falcon tube (Sarstedt AG and Co., KG, 51588 Nümbrecht, Germany) using the passive drool method. Samples were stored at or below −20 °C to −80 °C within a maximum of two hours. Prior to ABO antibody testing, the thawed samples were centrifuged at 4 °C until cell debris was removed and clear saliva was present (20 min at 20,000× *g*, or 15 min at 2500× *g*, collected in Falcon tubes or in Salimetric tubes, respectively).

The Ab196263 Human IgA Simple Step ELISA Kit (Abcam, Cambridge CB2 0AX, UK) was used for the quantitative measurement of total IgA^sal^ protein with 0.25 ng/mL sensitivity.

### 2.4. ABO Antibody Analysis

ABO blood type data were obtained from our patient and donor database or genotyped as previously described [6].

The samples were quantified for anti-A and anti-B classes by flow cytometry on a FACS Canto II (BD Biosciences, Heidelberg, Germany). Human red blood cells of blood types A1, B and O (all of them typed ccddee and Kell negative) were suspended in PBS (0.1%) and used for the binding of ABO antibodies present in serum and saliva. For the testing in serum, 25 µL of serum samples and 25 µL of 0.1% fixed RBCs (treated with Karnovsky’s fixative) were incubated [33].

Immunoglobulin-class-specific secondary antibodies labelled with Alexa Fluor™ 647 (fab fragment goat anti-human IgG, Fc_γ_-specific), R-phycoerythrin (fab fragment goat anti-human IgM, Fc_5µ_-specific) and Alexa Fluor 488 [F(ab’)_2_ goat anti-human IgA, α chain-specific)] were used (Jackson Immuno Research, West Grove, PA, USA). Specific antibody binding was detected as the median fluorescence intensity (MFI). Serum and saliva samples of type AB individuals were used as negative controls. To compare the values of antibody binding, anti-A/B levels were defined by geometric mean fluorescence intensity ratios, which were calculated by dividing the MFI of the sample of interest with the mean MFIs of the negative controls (*n* = 3 for serum; *n* = 5 for saliva).

### 2.5. Statistical Analysis

Continuous variables were summarized as median and first (Q1) and third (Q3) quartiles, and categorical variables as absolute and relative frequencies. Group differences between continuous variables were determined by the nonparametric Wilcoxon rank-sum test.

The anti-A/B values were not normally distributed and were log-transformed (logarithm with base 10) to meet the normality assumption. For statistical analysis, we computed two linear mixed effects models of the serum data, one for each dependent measure (anti-A and anti-B levels). We entered six fixed effects: Age, sex (reference: female), time point (reference: visit 1), antibody class (IgG as reference level), group (COVID-19 convalescents as reference group vs. serum controls) and two blood type categories (blood types O and B (reference group) for anti-A; blood types O and A (reference group) for anti-B). We further added a random intercept for each participant to account for inter-individual variability in anti-A/B levels.

As to the saliva data, there was a limited number of observations in saliva available for the second time point; we thus included only data collected during the first time point. Because of the very low values and variability of IgG and IgM measured, we focused on IgA levels. Anti-A and anti-B levels were investigated in a multiple linear regression model including age, sex, (reference: female) group (COVID-19 convalescents as reference group) and blood type (blood types O and B (reference group) for anti-A; blood types O and A (reference group) for anti-B) as predictors.

The associations between Anti-A/Anti-B IgA levels in the serum and saliva of the COVID-19 convalescents, Anti-A/Anti-B IgA levels and total IgA^sal^ in saliva, as well as between Anti-A/Anti-B and SARS-CoV-2-specific antibody concentrations were computed with Spearman correlation coefficients, stratifying by medium, time point, antibody class, blood type and group (COVID-19 convalescents vs. controls). A *p* value ≤ 0.05 was considered significant. All analyses were conducted using R version 4.1.0 (https://www.r-project.org), R Core Team, Vienna, Austria.

## 3. Results

### 3.1. Study Participants

The median time for the baseline visit of COVID-19 convalescent individuals after COVID-19 diagnosis (V1) was 1.5 months (44 days, IQR: 30–61), and the median time between V1 and V2 was approximately 2 months (66 days; IQR: 62–74). Five observations were censored at V1 and thirty-three were censored at V2 due to vaccination against SARS-CoV-2 to rule out a possible confounding effect of the vaccination on the ABO antibody levels. The median age of the COVID-19 convalescents was 39 years (IQR: 30–49) and 40% were male. Age and sex did not differ between the COVID-19 convalescents and the blood donors providing serum control samples. The saliva donors recruited were slightly younger (median: 34; IQR: 26–46) than the COVID-19 convalescents and included more males than females (60% male).

The distribution of the ABO blood types did not differ among the three groups analysed and is largely consistent with data reported for Europeans or Caucasians [34,35].

The characteristics of the study participants are reported in Table 1.

### 3.2. ABO Antibodies

**Figure 1:**
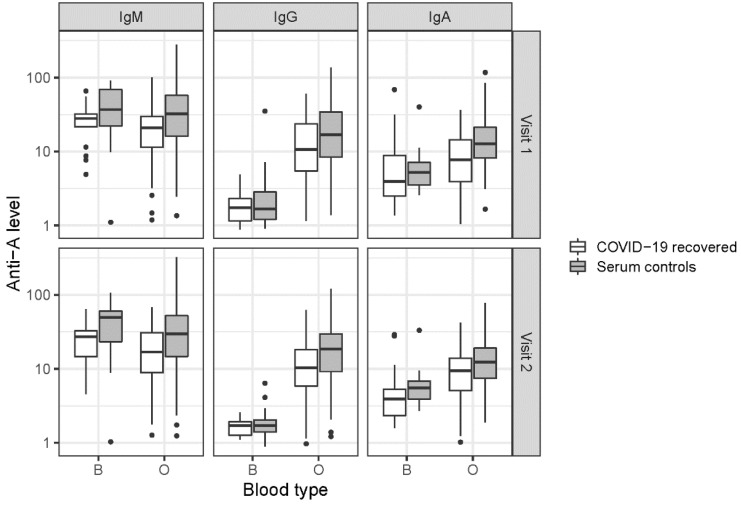
Anti-A levels in the serum of COVID-19 convalescents and controls. ABO Anti-A values of the isotypes IgM, IgG and IgA determined are indicated, each for ABO blood types B and O. Values are displayed on a log10-scale on the *y*-axis.

**Figure 2:**
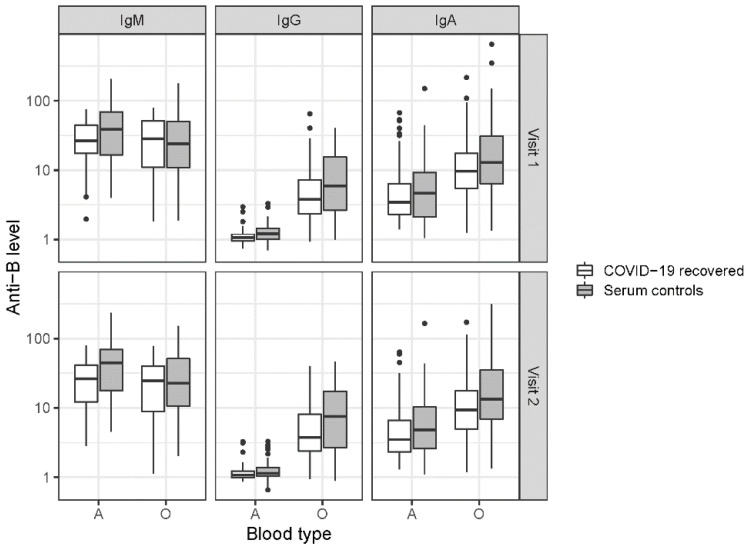
Anti-B levels in the serum of COVID-19 convalescents and controls. ABO Anti-B values of the isotypes IgM, IgG and IgA determined are indicated, each for ABO types A and O. Values are displayed on a log10-scale on the *y*-axis.

Study participants who previously tested positive for SARS-CoV-2 infection yielded significantly lower ABO Anti-A and Anti-B levels in the serum than controls (*p* < 0.001). As indicated in Table 2 and Figure 1 and Figure 2, the ABO antibody class analysis revealed the highest values in IgM, followed by IgA and IgG (*p* < 0.001). In Table 3 the results of the linear mixed effect models are displayed.

Anti-A levels were generally higher in subjects with blood type O than in those with blood type B (estimate: 0.82, *p* < 0.001). The difference between blood types was significant in IgA and IgG (*p* < 0.001), whereas it was not significant in IgM (*p* = 0.126). The difference between blood types was larger in IgG than IgA. There was no significant effect of age (*p* = 0.976) or sex (*p* = 0.444) and no statistically significant difference between time points (*p* = 0.273).

Consistent with the pattern in anti-A, anti-B levels (Table 2 and Figure 2) were highest in IgM, followed by IgA and IgG (*p* < 0.001). Blood groups interacted significantly with the antibody class: levels were significantly higher in blood group O vs. A (estimate: 0.65, *p* < 0.001) in IgG and in IgA (*p* < 0.001). In IgM, levels were higher in blood group A vs. O (*p* = 0.001). For anti-B levels, the effect of sex was significant (*p* = 0.011), revealing generally lower levels in men than in women. Again, there was no statistically significant difference between age (*p* = 0.618) and time points (*p* = 0.786).

ABO antibody levels tested in saliva of COVID-19 convalescents did not differ significantly from antibody levels tested in saliva controls (anti-A: *p* ≥ 0.525; anti-B: *p* ≥ 0.338). Results of multiple regression models for saliva samples are reported in the supplementary materials (Tables S1 and S2, and Figure S1).

**Figure 3:**
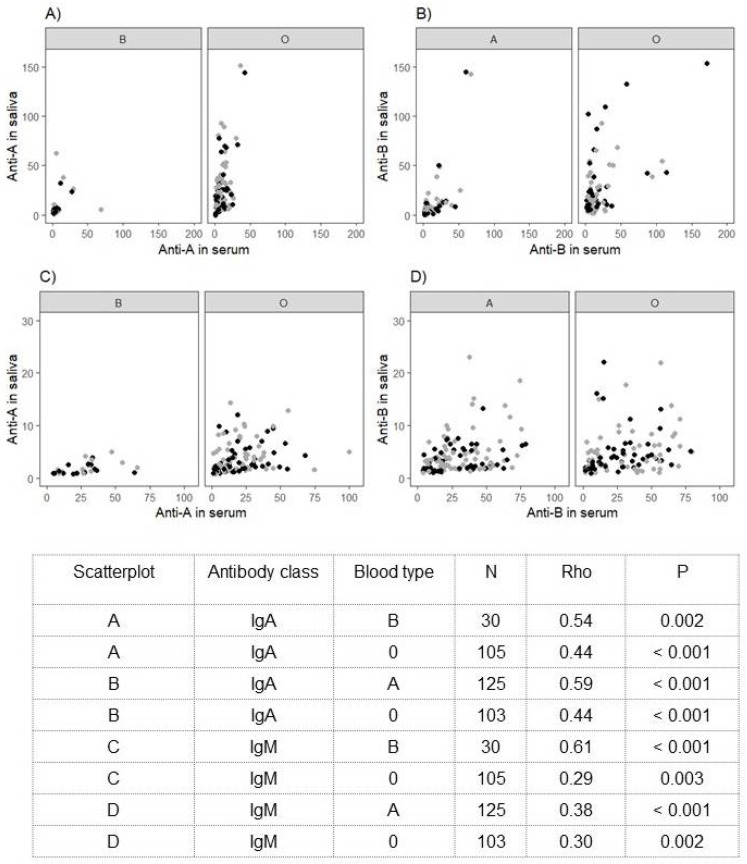
(**A**–**D**). Correlation of ABO antibodies in the serum and saliva of COVID-19 convalescents. Scatterplots A and B for class IgA; scatterplots C and D for class IgM; note that observations at visits 1 and 2 are gathered together. N, number of individuals.

We observed correlations for anti-A/B IgA (Rho: 0.44–0.59) and IgM (Rho; 0.29–0.61) between serum and saliva samples in the participants who previously tested positive for SARS-CoV-2. Scatterplots and correlations are indicated in Figure 3A–D.

Some of the studied individuals (*n* = 21) had anti-A and/or anti-B levels higher than 140 in serum and/or saliva (Anti-A: 144.1–321.2; Anti-B: 142.4–653.2; including visit 1 and visit 2). The values are indicated in Supplementary Table S3.

Of these, 18 (85.7%) were participants from the serum or saliva control groups, 12 (57.1%) had blood type O, 9 (42.9%) had blood type A, and 14 (66.7%) were women.

### 3.3. Total IgA in Saliva

The concentration of total IgA^sal^ antibodies was lower in the group of COVID-19 convalescents as compared with the saliva controls (V1: Convalescents, median 28.3, IQR: 19.8–40.2, *n* = 126; saliva controls, median 32.5, IQR: 20.7–48.6, *n* = 162; V2: Convalescents, median 23.6, IQR: 16.0–32.8, *n* = 120; saliva controls, median 30.0, IQR: 24.1–41.3, *n* = 27). Differences were significant at V1 and V2 (*p* = 0.038 and *p* = 0.027, respectively). Note that at V2, only 27 of 153 (18%) saliva controls were available for IgA^sal^ analysis (Supplementary Figure S2).

**Figure 4:**
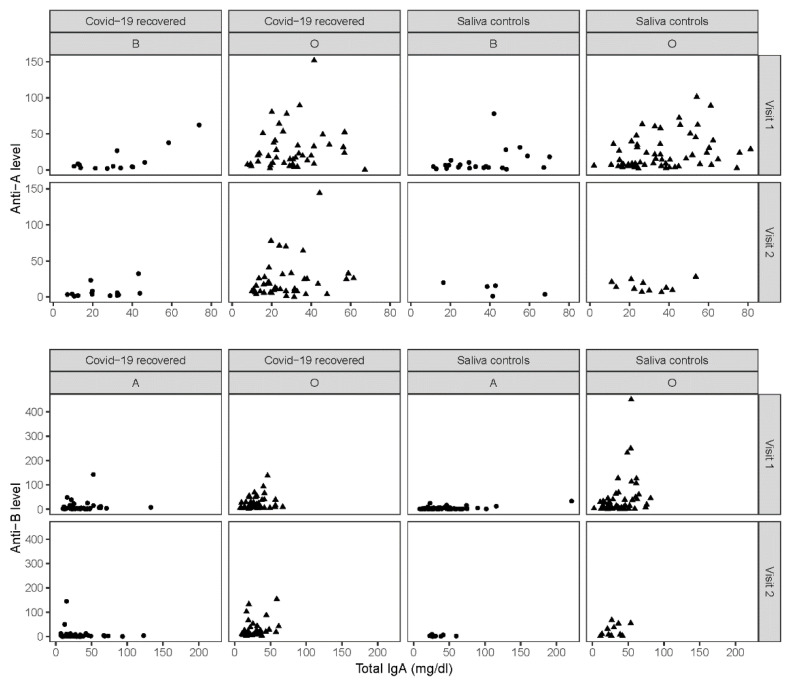
ABO Anti-A- and anti-B-specific IgA analysed in relation to total IgA in saliva. Observations for types A and B are depicted as circles, and those for blood type O are depicted as triangles.

The relationship between total IgA^sal^ concentrations and anti-A/B IgA levels in saliva is displayed in Figure 4. Spearman correlations were not significant, with the only exception being the significant association between total IgA^sal^ and anti-A/B levels in saliva controls with blood type O at V1 (anti-A: rho = 0.30, *p* = 0.024, *n* = 58; anti-B: rho = 0.35, *p* = 0.007, *n* = 58).

### 3.4. ABO Antibodies and SARS-CoV-2-Specific Antibody Concentrations

In the COVID-19 convalescents, Spearman correlations between ABO antibodies and virus-specific total antibody concentrations revealed no significant associations (−0.44 < rho < 0.42, *p* > 0.053). The longitudinal development of pan-Ig antibody concentrations in this cohort has already been described [32].

## 4. Discussion

We detected significantly lower levels of anti-A and anti-B antibodies of classes IgM, IgG and IgA in the serum of patients with a previous diagnosis of mild COVID-19 than in a control group of blood donors who had never been infected with SARS-CoV-2. This observation confirms and extends the results obtained by Deleers et al. [10], demonstrating lower IgM anti-A and anti-B serum levels in COVID-19 patients than in controls.

Interestingly, we found no significant differences between the ABO antibody levels in the saliva of cases and controls. In parallel, the concentration of total IgA in saliva was approximately reduced by 10–20% in the patients compared with the controls, as it might be expected in mild coronavirus disease. Previously, specific IgA against the SARS-CoV-2 spike protein has been shown to appear early in infected patients [36], and increased total IgA in serum was associated with severe COVID-19 [37]. However, the dynamic of total IgA in saliva as a part of the immune response and its role in COVID-19 needs to be elucidated by further studies.

As there are obvious differences in ABO antibody levels in the serum, we observed only a weak correlation for ABO IgA between serum and saliva samples in the group who were previously infected with SARS-CoV-2. Critical discussion and support for additional research should be given to the assumption that an effect of isoagglutinins may be attributed only to antibodies present in blood serum but does not refer to the respiratory tissue.

In agreement with previous results [38,39], we did not observe a correlation between the antibody response to SARS-CoV-2 and ABO antibodies, such as high anti-A levels. This observation may be consistent with the theory that the humoral immune response to the virus is somewhat correlated with the virulence of the pathogen as contributor to the severity of the disease [40], although the evidence for the relationship between ABO and disease severity is still ambiguous [6,41,42].

Since SARS-CoV-2 infection does not always lead to the appearance of disease-related symptoms in an individual, the initial entry phase of infection must be distinguished from the progression of infection and the outbreak of the disease. In susceptible or infected individuals, the potential interaction of ABO antibodies with the virus may result in some form of protection or a milder course of disease. Likewise, COVID-19 was associated with the levels of anti-carbohydrate anti-Tn antibodies that also recognize α-*N*-acetylgalactosamine [43]. ABO antibody titres were also discussed as a potential surrogate marker, which might reflect the constitution of an individual’s humoral immune system. However, this might depend upon other factors, such as continuous antigenic stimulation, and remains a controversial issue [44].

ABO antibodies may be adsorbed or consumed by viral particles during infection which could also explain why COVID-19 convalescents’ antibody levels were lower than those of controls [10]. A potential increase in or booster of anti-A levels in response to SARS-CoV-2 infection may be anticipated when group A antigen has been incorporated into the S protein structure of SARS-CoV-2. The low anti-A/anti-B levels in the COVID-19 convalescents detected shortly after the acute phase of infection and up to five months after infection contradict this theory. A higher susceptibility to SARS-CoV-2 infection because of individually pre-existing low ABO antibody levels facilitating virus attachment to host cells, rather than other dynamic changes in response to the infection, may be suggested. Accordingly, Wasiluk et al. failed to find an increase in anti-A IgM titres in platelet concentrates from blood group O donors as determined before and after infection with the virus [45].

However, we identified some individuals who had significantly higher anti-A/anti-B levels than others. ABO antibodies may have been increased in cases of ABO-incompatible pregnancies, affecting mostly female research participants with blood type O [14,46,47,48]. Literature reporting on this issue is mainly related to ABO isoagglutinins of the IgG isotype. Given that some microorganisms can stimulate antibodies to blood group antigens, these individuals may have had other infections at the time of sampling, or the antibodies could have been induced by consuming probiotics [49]. In addition, physiological variations in anti-A and anti-B titres between individuals of the same blood type, including individuals with low- and high-titre isoagglutinins, have been described [19,20,49,50]. Information regarding the presence of infectious disorders other than SARS-CoV-2, probiotic use at the time of sampling and a history of an ABO-incompatible pregnancy were not evaluated either in patients or in controls. Importantly, our study results were not affected by the exclusion of these samples in the statistical analyses.

The proportions of individuals with the various ABO types did not differ significantly between the cohort of individuals who recovered from mild COVID-19 and the healthy reference population. It is well described that the progression of the disease also depends on more relevant risk factors [51], and blood type O was associated with a lower risk for venous thromboembolism and cardiovascular disease [52,53,54,55]. As observed in our previous study, blood type O may therefore offer protection against hospitalization, but not against a mild or asymptomatic course of COVID-19.

Some limitations of this study should be kept in mind. Since the serum and saliva samples came from different blood donors, the relationship of these two sources could only be investigated in the group of COVID-19 convalescents but not in our controls. Further, the majority of control subjects who donated saliva had been vaccinated against SARS-CoV-2 by the time they attended the second saliva sampling. The exclusion of their second samples resulted in a small sample size for donation 2.

Taken together, our results support the idea that ABO antibodies play a protective role against SARS-CoV-2 infection. As a proxy for mucosal immunity in the respiratory tract of affected individuals, our unexpected observation of significantly reduced antibody levels only in serum but not in saliva remains insufficiently understood. Further in vitro studies are urgently needed to clarify the molecular mechanism that explains the contribution of ABO antibodies to the pathogenesis of SARS-CoV-2 infection.

## Figures and Tables

**Table 1:** Characteristics and demographics of study participants.

	COVID-19 Convalescents (N = 187)	Serum Controls (N = 195)	*p* (Convalescents vs. Serum Controls)	Saliva Controls (N = 164)	*p* (Convalescents vs. Saliva Controls)
Age Median (Q1, Q3)	39.0 (30.0, 49.0)	39.0 (30.0, 49.5)	0.806 *	34.0 (26.0, 46.0)	0.004 *
Sex			1.000 ^†^		<0.001 ^†^
Male	75 (39.9%)	78 (40.0%)		98 (59.8%)	
Female	112 (59.9%)	117 (60.0%)		66 (40.2%)	
ABO blood type			0.983 ^†^		0.576 ^†^
A	82 (43.9%)	85 (43.6%)		66 (40.2%)	
AB	18 (9.6%)	17 (8.7%)		18 (11.0%)	
B	17 (9.1%)	17 (8.7%)		22 (13.4%)	
O	70 (37.4%)	76 (39.0%)		58 (35.4%)	
Days between COVID-19 diagnosis and first sampling (V1) Median (Q1, Q3)	44.0 (30.0, 61.0)	NA	NA	NA	NA
Days between samplings 1 and 2 Median (Q1, Q3)	66.0 (62.0, 74.0)	64.0 (62.0, 72.0)	0.125 *	56.0 (56.0, 60.0)	<0.001 *

N, number of individuals; NA, not applicable; Q1, first quartile; Q3, third quartile. * Wilcoxon rank-sum test; ^†^ Fisher’s exact test.

**Table 2:** Anti-A and Anti-B levels in the serum of COVID-19 convalescents and controls.

			COVID-19 Convalescents		Serum Controls	
Anti-A	Blood Type	Visit	N	Median (Q1, Q3)	N	Median (Q1, Q3)
IgM	B	1	17	27.9 (21.6, 32.3)	17	36.9 (22.1, 68.7)
IgM	B	2	16	27.1 (14.7, 32.6)	17	49.4 (23.3, 60.6)
IgM	O	1	70	21.0 (11.4, 29.8)	76	32.5 (16, 57.4)
IgM	O	2	58	16.9 (8.9, 30.7)	76	29.6 (14.7, 52.1)
IgG	B	1	17	1.7 (1.2, 2.3)	17	1.7 (1.2, 2.8)
IgG	B	2	16	1.7 (1.3, 1.9)	17	1.7 (1.4, 2)
IgG	O	1	70	10.6 (5.5, 23.7)	76	16.9 (8.4, 34.1)
IgG	O	2	58	10.4 (5.8, 18.3)	76	18.6 (9.2, 29.6)
IgA	B	1	17	3.9 (2.5, 8.8)	17	5.2 (3.5, 7.1)
IgA	B	2	16	3.9 (2.4, 5.5)	17	5.5 (3.9, 6.8)
IgA	O	1	70	7.7 (3.9, 14.4)	76	12.7 (8.2, 21.3)
IgA	O	2	58	9.5 (5.1, 14)	76	12.3 (7.4, 19)
Anti-B						
IgM	A	1	82	26.5 (17.5, 44.3)	85	38.8 (16.5, 69.3)
IgM	A	2	63	26.2 (12.2, 41.2)	85	44.6 (17.8, 69)
IgM	O	1	70	28.3 (11, 51.4)	76	24.1 (10.8, 50.5)
IgM	O	2	58	24.5 (8.9, 40)	76	22.5 (10.6, 51.2)
IgG	A	1	82	1.1 (0.9, 1.2)	85	1.2 (1, 1.4)
IgG	A	2	63	1.1 (1, 1.2)	85	1.1 (1, 1.4)
IgG	O	1	70	3.8 (2.3, 7.2)	76	5.9 (2.6, 15.5)
IgG	O	2	58	3.8 (2.4, 8.1)	76	7.5 (2.7, 17.3)
IgA	A	1	82	3.4 (2.3, 6.3)	85	4.6 (2.1, 9.3)
IgA	A	2	63	3.5 (2.3, 6.6)	85	4.8 (2.6, 10.3)
IgA	O	1	70	9.6 (5.4, 17.5)	76	12.8 (6.4, 31)
IgA	O	2	58	9.3 (5, 17.5)	76	13.4 (6.8, 35.2)

N, number of individuals; statistical analysis of this data is included in Table 3.

**Table 3:** Results of linear mixed effects models for anti-A and anti-B in the serum of COVID-19 convalescents versus controls.

	Estimate	95% CI	*p*
Anti-A			
Age	0.0001	(−0.003; 0.003)	0.976
Sex (male)	0.03	(−0.05; 0.11)	0.444
Time point (visit 2)	−0.02	(−0.06; 0.02)	0.273
Antibody_class: IgM	1.16	(1.05; 1.28)	<0.001
Antibody_class: IgA	0.46	(0.35; 0.58)	<0.001
Group: Serum controls	0.19	(0.11; 0.26)	<0.001
Blood type: O	0.82	(0.7; 0.94)	<0.001
Antibody_class × blood type (IgM:O)	−0.94	(−1.06; −0.81)	<0.001
Antibody_class × blood type (IgA:O)	−0.57	(−0.69; −0.44)	<0.001
Anti-B			
Age	−0.001	(−0.003; 0.002)	0.618
Sex (male)	−0.08	(−0.14; −0.02)	0.011
Time point (visit 2)	−0.004	(−0.04; 0.03)	0.786
Antibody_class: IgM	1.4	(1.34; 1.45)	<0.001
Antibody_class: IgA	0.63	(0.58; 0.68)	<0.001
Group: Serum controls	0.10	(0.04; 0.16)	0.001
Blood type: O	0.65	(0.58; 0.73)	<0.001
Antibody_class × blood type (IgM:O)	−0.8	(−0.88; −0.73)	<0.001
Antibody_class × blood type (IgA:O)	−0.27	(−0.34; −0.19)	<0.001

CI, confidence interval.

## Data Availability

All of the analysed data is contained within the article or supplementary material. The raw data are available on request from the corresponding author.

## Supplementary Materials

The following supporting information can be downloaded at: https://www.mdpi.com/article/10.3390/jcm11154513/s1, Table S1: Anti-A and anti-B levels in the saliva of COVID-19 convalescents and controls; Table S2: Results of multiple regression models investigating the effect of age, sex, blood type and group on anti-A/B levels in the saliva of COVID-19 convalescents and controls; Table S3: Study participants exhibiting high anti-A/anti-B levels compared with other participants; Figure S1: Anti-A and anti-B values in the saliva of COVID-19 convalescents and controls; Figure S2: Total IgA concentration in saliva of COVID-19 convalescent individuals and controls.
