# Supplementary material for: Lower Levels of ABO Anti-A and Anti-B of IgM, IgG and IgA Isotypes in the Serum but Not the Saliva of COVID-19 Convalescents
Source: J Clin Med. 2022 Aug 2;11(15):4513. doi: 10.3390/jcm11154513 (PMC9369710; doi:10.3390/jcm11154513)
Supplement: Supplementary file 1 [file jcm-11-04513-s001.zip › jcm-1820592-supplementary.pdf]

Supplemental data

Table S1. Anti-A and anti-B levels in the saliva of COVID-19 convalescents and controls

				**COVID-19 convalescents**		**Saliva controls**
**Anti-A**	ABO blood type	Visit	N	Median (Q1, Q3)	N	Median (Q1, Q3)
IgM	B	Visit 1	15	1.8 (1.3, 2.8)	22	2.3 (1.9, 4.3)
IgM	B	Visit 2	15	1.3 (1, 2.1)	5	2.1 (1.8, 2.5)
IgM	O	Visit 1	56	3.8 (2.1, 5.8)	58	3.3 (1.9, 6.2)
IgM	O	Visit 2	49	2.6 (1.8, 4.6)	11	2.4 (1.7, 5)
IgG	B	Visit 1	15	1 (0.9, 1.1)	22	1 (1, 1.1)
IgG	B	Visit 2	15	1 (0.9, 1.1)	5	1 (1, 1.1)
IgG	O	Visit 1	56	1.2 (1, 1.6)	58	1.2 (1, 1.6)
IgG	O	Visit 2	49	1.1 (0.9, 1.6)	11	1.2 (1, 2.6)
IgA	B	Visit 1	15	5 (3.5, 9.4)	22	4.8 (3.2, 12.5)
IgA	B	Visit 2	15	4.1 (2.5, 5.9)	5	14.4 (3.8, 15.6)
IgA	O	Visit 1	56	15.7 (7.3, 34)	58	14.4 (6.8, 35.5)
IgA	O	Visit 2	49	12.9 (7.7, 25.7)	11	12.8 (9.2, 20.1)
**Anti-B**						
IgM	A	Visit 1	70	3.4 (2.3, 5.4)	66	3.1 (2.2, 4.6)
IgM	A	Visit 2	55	2.4 (1.7, 5.3)	9	3.6 (1.6, 5.1)
IgM	O	Visit 1	56	4 (2.3, 6.3)	58	3.6 (2.3, 6.2)
IgM	O	Visit 2	47	3.7 (2.2, 5.4)	11	3.4 (1.8, 4.3)
IgG	A	Visit 1	70	0.9 (0.8, 1)	66	1 (0.9, 1.1)
IgG	A	Visit 2	55	0.9 (0.7, 0.9)	9	1.1 (1, 1.1)
IgG	O	Visit 1	56	1 (0.9, 1.2)	58	1 (0.9, 1.3)
IgG	O	Visit 2	47	1 (0.9, 1.1)	11	1.2 (0.9, 1.3)
IgA	A	Visit 1	70	3.2 (1.8, 5.9)	66	2 (1.3, 4.9)
IgA	A	Visit 2	55	2.7 (1.7, 5)	9	2.2 (1.1, 3.4)
IgA	O	Visit 1	56	12.1 (6, 29.2)	58	13.1 (4.9, 39.5)
IgA	O	Visit 2	47	10.5 (8, 25.7)	11	9.8 (5.8, 45.4)

N, number of individuals; Interquartile range: Q1, first quartile and Q3, third quartile

Table S2. Results of multiple regression models investigating the effect of age, sex, blood type and group on anti-A/B levels in the saliva of COVID-19 convalescents and controls

**Anti-A level**	Estimate	95% CI	P
Age	0.002	(-0.005;0.009)	0.523
Sex: Male	-0.2	(-0.37;-0.03)	0.02
ABO blood type: O	0.36	(0.17;0.54)	< 0.001
Group: Saliva controls	0.06	(-0.12;0.23)	0.525
**Anti-B level**			
Age	0.003	(-0.003;0.008)	0.34
Sex: Male	-0.16	(-0.29;-0.02)	0.021
Blood group: O	0.66	(0.53;0.79)	< 0.001
Group: Saliva controls	-0.07	(-0.2;0.07)	0.338

CI, confidence interval; No significant differences between the study samples of the COVID-19 convalescents and the controls were found.

Table S3. Study participants exhibiting high anti-A/anti-B levels compared to other participants

Group	ABO blood type	Sex	Age	Specimen	Antibody class	Anti-A level visit 1	Anti-A level visit 2	Anti-B level visit 1	Anti-B level visit 2
COVID-19 convalescent*	O	Female	41	Saliva	IgA	49.0	32.6	137.9	153.4
COVID-19 convalescent*	O	Female	41	Serum	IgA	14.8	14.0	216.3	171.4
COVID-19 convalescent	O	Female	52	Saliva	IgA	151.7	144.1	65.6	87.3
COVID-19 convalescent	A	Female	36	Saliva	IgA	0.3	0.9	142.4	145.0
Control	O	Female	45	Serum	IgA	117.1	2.2	653.2	1.6
Control	O	Female	32	Serum	IgM	76.8	321.2	15.2	24.5
Control	A	Male	40	Serum	IgM	1.1	1.0	117.9	161.6
Control	A	Female	36	Serum	IgM	1.3	1.2	206.3	193.1
Control	A	Female	41	Serum	IgA	1.3	1.3	149.9	164.9
Control	A	Female	45	Serum	IgM	0.9	0.9	19.1	233.2
Control	A	Male	21	Serum	IgM	1.1	1.1	107.8	143.8
Control	O	Female	42	Serum	IgA	9.5	8.7	349.0	312.0
Control	A	Male	59	Serum	IgM	1.1	1.0	178.7	152.2
Control	O	Male	29	Serum	IgM	51.1	43.9	177.2	151.8
Control	O	Female	29	Serum	IgM	277.2	240.0	78.2	65.1
Control	O	Female	29	Serum	IgM	180.9	273.8	52.5	61.1
Control	O	Male	42	Serum	IgA	10.9	10.6	147.6	143.9
Control	A	Male	30	Serum	IgM	0.9	2.5	151.5	165.9
Control	A	Female	27	Serum	IgM	1.1	1.1	142.5	131.5
Control	O	Female	38	Saliva	IgA	101.3	NA	450.2	NA
Control	O	Female	25	Saliva	IgA	15.6	NA	231.6	NA
Control	O	Male	28	Saliva	IgA	45.2	NA	249.4	NA

ABO antibody levels were measured in serum and saliva samples collected at two time points (visit 1: baseline visit; visit 2: two months later) in individuals who recovered from COVID-19 and in controls who had never been infected with SARS-CoV-2. NA, not available; *Serum and saliva samples belong to the same participant.

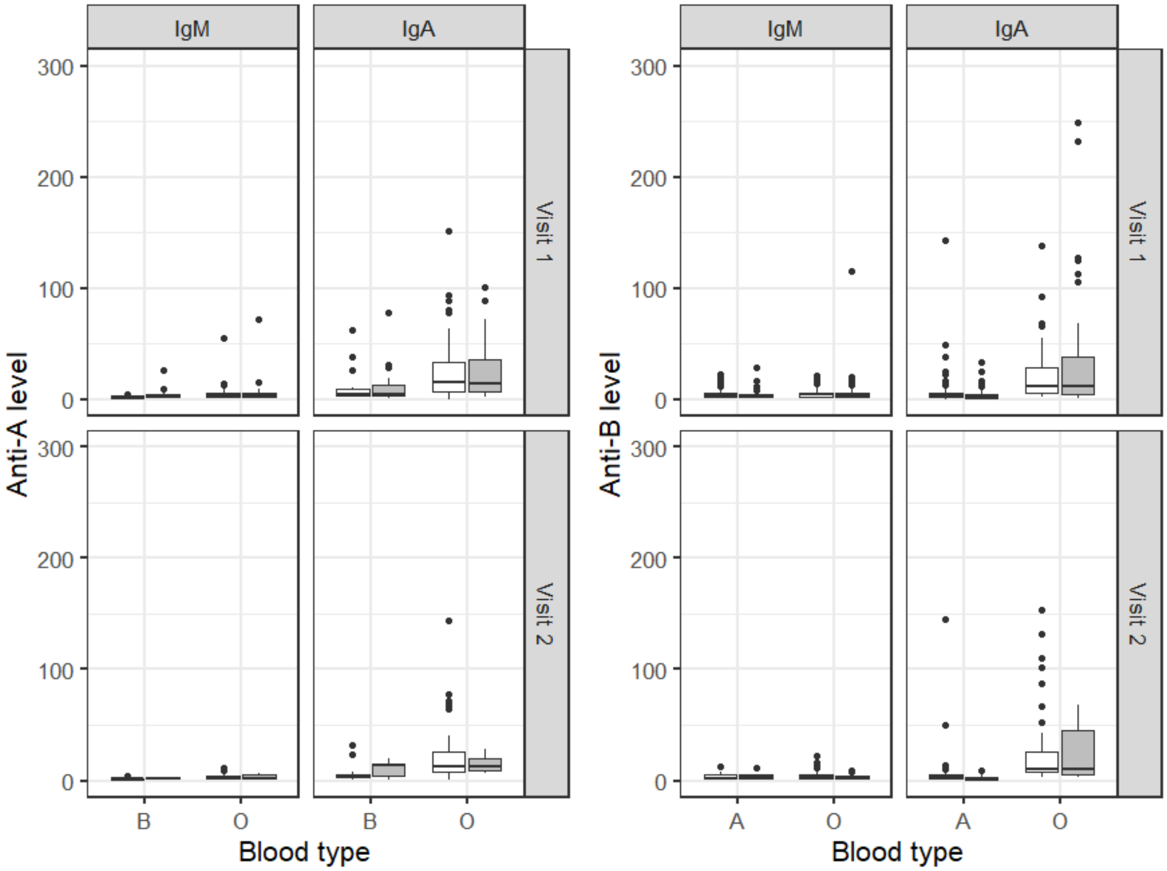

Figure S1.

Figure S1. Anti-A and anti-B values in the saliva of COVID-19 convalescents and controls. ABO antibody levels measured at two time points (visit 1: baseline visit and visit 2: two months later) are shown. Participants who recovered from COVID-19 are displayed in white, and healthy blood donors are displayed in gray. Only IgM and IgA are reported because values for IgG were extremely low. There was no significant difference between antibody levels detected in COVD-19 convalescents and controls. Note that one observation with an anti-B level > 400 is not shown in the plot (IgA, visit 1).

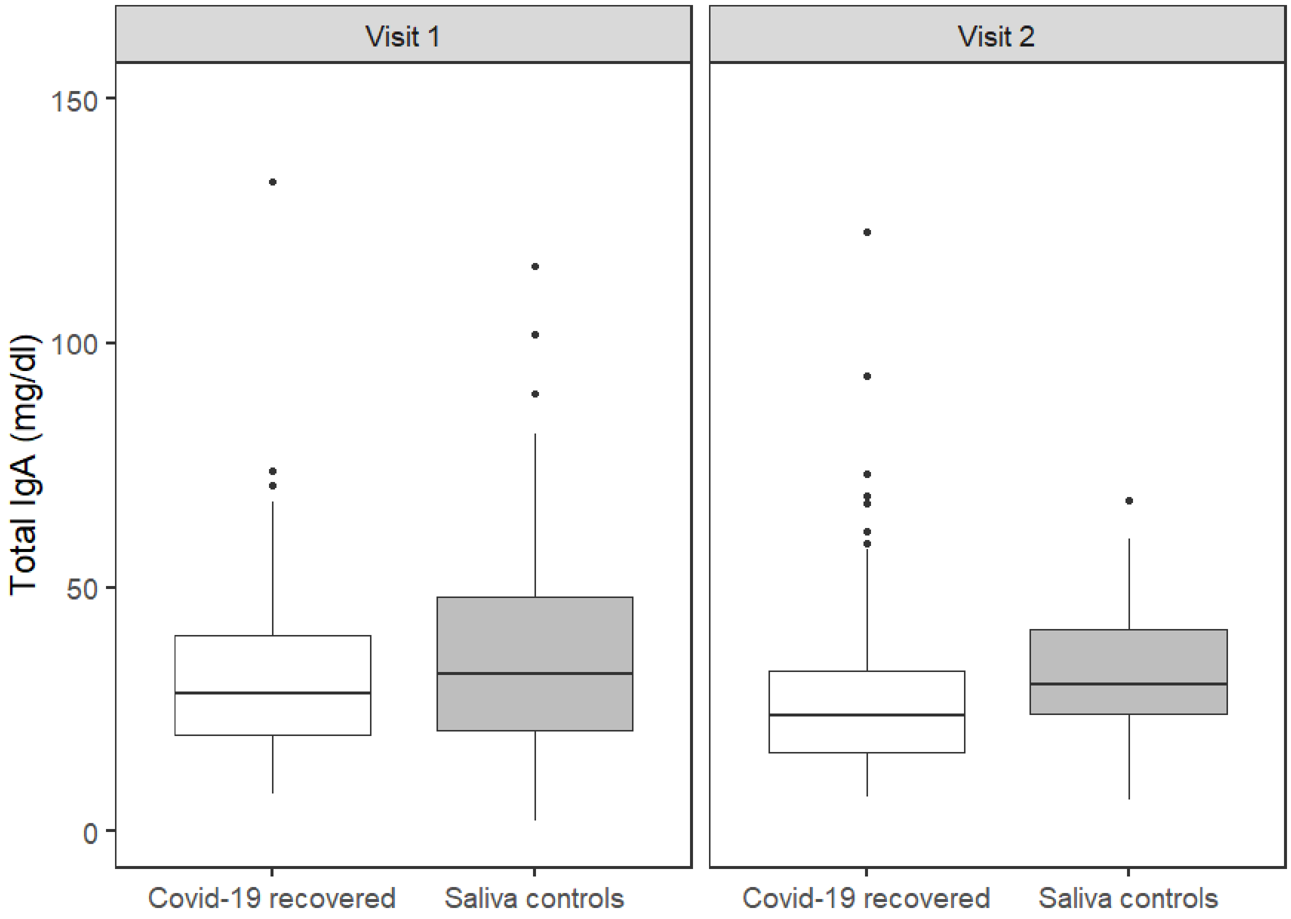

Figure S2. Total IgA concentration in saliva of COVID-19 convalescent individuals and controls. Values measured in saliva samples collected at two time points (visit 1: baseline visit and visit 2: two months later) are indicated. Differences were significant at visit 1 [p.0.038 (COVID-19 recovered n=126, median 28.3, IQR 19.8-40.2; saliva controls: n=162, median 32.5, IQR 20.7-48.6)], and at visit 2 [p=0.027 (COVID-19 recovered: n=120, median 23.6, IQR 16.0-32.8; saliva controls: n=27, median 30.0, IQR 24.1-41.3)]. Note that two observations at visit 1 for saliva controls are not shown (level > 150 mg/dl) and hat at visit 2, only 27 of 153 (18%) saliva controls were available for IgA analysis in saliva.
